# Clinicopathological and Molecular Analysis of Aqueous Humor for the Diagnosis of Feline Infectious Peritonitis

**DOI:** 10.3390/vetsci11050207

**Published:** 2024-05-09

**Authors:** Angelica Stranieri, Stefania Lauzi, Saverio Paltrinieri

**Affiliations:** Department of Veterinary Medicine and Animal Sciences (Divas), University of Milan, Via dell’Università 6, 26900 Lodi, Italy; angelica.stranieri@unimi.it (A.S.); stefania.lauzi@unimi.it (S.L.)

**Keywords:** ocular fluids, feline coronavirus, FIP, cytology, FCoV PCR, ocular proteins, diagnostic accuracy

## Abstract

**Simple Summary:**

The aim of this study was to evaluate the utility of clinicopathological and molecular findings on aqueous humor (AH) for the diagnosis of feline infectious peritonitis (FIP), a disease sustained by the feline coronavirus (FCoV). To this aim, we investigated the presence of viral genome or of cells potentially consistent with inflammation and the protein content in AH samples collected from cats with or without FIP. The results of this study demonstrated that although none of these tests were sensitive or specific enough to be used alone to diagnose FIP, the detection of inflammatory cells in the AH, especially if associated with positive PCR, may work as a supportive test when the probability of FIP is high, based on the additional information provided via physical examination or laboratory tests. Additionally, the concentration of intraocular protein in cats with FIP was very high, especially in the non-effusive form. However, the diagnostic role of protein measurement in ocular fluids needs to be further assessed in future studies.

**Abstract:**

Background: This study was designed to assess the diagnostic utility for FIP of cytology, protein measurement and RT-PCR for feline coronaviruses (FCoV) on aqueous humor (AH), since little information is currently available. Methods: AH samples (*n* = 85) were collected post-mortem from 13 cats with effusive FIP (E-FIP), 15 with non-effusive FIP (NE-FIP) and 16 without FIP, to perform cytology (*n* = 83) and RT-PCR (*n* = 66) and to calculate their sensitivity, specificity and positive and negative likelihood ratios (LR+ and LR−). The protein concentration was measured on 80 fluids. Results: The proportion of RT-PCR positive samples did not differ among groups, while positive cytology was more frequent in samples with FIP (*p* = 0.042) or positive RT-PCR (*p* = 0.007). Compared with other groups, the protein concentration was higher in samples with NE-FIP (*p* = 0.017), positive RT-PCR (*p* = 0.005) or positive cytology (*p* < 0.001). The specificity of cytology together with RT-PCR, cytology alone, RT-PCR alone and cytological proteinaceous background were 90.0%, 84.6%, 70.0%, 61.5%, and the LRs 3.48, 2.65, 1.83, 1.64, respectively. However, their sensitivities were low (34.8–63.0%) and their LR− high (0.60–0.72). Conclusions: Based on the LR+, cytology and/or RT-PCR may support the diagnosis when the pre-test probability of FIP is high. The concentration of intraocular protein is a promising marker, especially in NE-FIP.

## 1. Introduction

Feline coronavirus (FCoV) is a ubiquitous virus which usually causes either asymptomatic infections or mild intestinal disorders [1]. In a low percentage of infected cats, viral mutations and an inadequate immune response of the hosts lead to feline infectious peritonitis (FIP) [1]. FIP arises with two clinical presentations, the effusive and the non-effusive form. Neurological and ocular signs are common especially in non-effusive FIP (NE-FIP), with a prevalence of 60% in affected cats, but ocular involvement may actually be underestimated [2,3]. Moreover, FIP is described as one common cause of feline uveitis and it was found to be the most common cause of uveitis in a cohort of 120 cats [4,5,6]. Ocular lesions in FIP usually appear as pyogranulomatous uveitis, but posterior involvement with chorioretinitis and retinal vasculitis is also described [5]. Changes in the color of the iris and keratic precipitates in the anterior chamber are commonly seen [3].

Effusion analyses along with history and other laboratory compatible results can help to obtain a high index of suspicion for FIP [1]. On the other hand, the diagnosis of FIP in the non-effusive form can be achieved only with invasive and risk-associated procedures aimed to collect bioptic samples, since confirmatory diagnosis can be obtained only with histopathological exams [1,7,8,9].

Aqueocentesis is used in veterinary medicine with minimal risk, and aqueous humor (AH) cytology is useful in the diagnosis of neoplastic diseases, particularly lymphoma, in dogs and cats [10,11]. Recently, the immunocytochemical analysis (ICC) of aqueous humor was investigated as a diagnostic tool for FIP and showed a sensitivity of 64% and a specificity of 81.8%, showing that it cannot be used as a single confirmatory test [12,13].

Only a few studies have evaluated RT-PCR on AH for FIP, either on few samples or without including cats with diseases other than FIP in the study, showing a low sensitivity [13,14,15,16]. Moreover, data regarding the clinicopathological characteristics of AH during FIP are scarce, as cytological examination is described only in a few cases, while information regarding AH protein content is lacking [10]. Finally, most of the studies on the diagnostic value of AH analysis for the diagnosis of FIP are focused on the detection of abnormalities in ocular fluids from eyes clinically or pathologically affected by lesions typical of FIP. Our hypothesis is that the systemic vasculitis that characterizes the pathogenesis of FIP may induce the leakage of FCoV into the AH, with a subsequent inflammation and increase in intraocular proteins, irrespective of the presence of clinically evident or pathologically detectable ocular lesions. Thus, the aims of this study were the following: (1) to assess the frequency of ocular samples with positive RT-PCR for FCoVs and with clinico-pathological changes potentially consistent with inflammation (increased intraocular protein concentration and peculiar cytological findings) in cats with FIP, irrespective of the clinical presentation and the presence of an ocular disease, compared with the frequency of the same changes in cats with diseases other than FIP; (2) to assess the diagnostic utility of clinicopathological (cytological examination, protein content measurement) and molecular tests (reverse transcriptase PCR—RT-PCR—for FCoV) on aqueous humor samples for the diagnosis of FIP, by evaluating specificity, sensitivity and likelihood ratios in cats with and without FIP that, irrespective of the presence of ocular lesions, have positive molecular or cytological results in at least one of the two eyes.

## 2. Materials and Methods

### 2.1. Caseload

Aqueous humor samples were collected post-mortem at the Veterinary Teaching Hospital of Milan from cats deceased or euthanized with a suspected case of FIP or due to other conditions and were subjected to necropsy for diagnostic purposes. All the above methods were performed within routine diagnostic procedures and after the owners’ signing of an informed consent about the use of residual amounts of tissues and/or samples for research purposes. Therefore, according to the Ethical Committee of the University of Milan (decision n° 2, 2016), no additional formal request of authorization to the Ethical Committee were needed.

The inclusion criteria were the following: (1) the possibility of collecting and processing AH humor samples immediately after death; (2) the availability of clinical information and the results of post-mortem examinations, i.e., necropsy and histology on abdominal and/or thoracic organs, depending both on the presence of gross lesions and on diagnostic suspect, followed by anti-FCoV immunohistochemistry in order to classify the cats as affected or not affected by FIP.

### 2.2. AH Collection and Processing

AH samples were obtained through limbal entry and anterior chamber paracentesis, using a 25 g needle on a 1-mL syringe, which was inserted parallel to the iris and between this latter and the cornea [11]. AH was gently aspirated, with volumes varying between 0.3 mL and 0.5 mL, depending on the age of the cat and the eyes’ size, and these were immediately collected in pediatric volume (0.5-mL) EDTA tubes.

Fifty µL of AH were cytocentrifuged for 5 min at 450× rpm (Shandon, Pittsburgh, PA, USA). The obtained slide was stained with May–Grunwald–Giemsa and used for cytological examination.

The remaining AH aliquot was centrifuged (3500× *g*, 5 min) and the obtained supernatant and pellet were frozen at −20 °C upon use for protein content measurement and RT-PCR for FCoV, respectively. When pellets were not macroscopically visible, the amount of fluid remaining after the removal of the minimum volume of supernatant needed for protein content measurement (approximately 150 µL) was harvested and frozen to be used for RT-PCR.

### 2.3. Cytological Examination

Each cytocentrifuged slide was evaluated for cellularity, blood contamination or contamination with melanin or corneal tissue. The presence of inflammatory cells was considered consistent with FIP, while acellular samples or samples characterized by very scarce cellularity or by the presence of mononuclear cells alone were considered not consistent with FIP. The presence of a granular, eosinophilic proteinaceous background was also recorded and considered as positive (i.e., consistent with FIP) [10].

### 2.4. Measurement of AH Protein Content

On frozen-thawed AH supernatant samples, protein content was measured using the automated spectrophotometer BT3500 (Biotecnica Instruments, Rome, Italy) and the pyrogallol red-molybdate method (Urinary Protein-L, Biotecnica Instruments, Rome, Italy). This method was preferred to the biuret method usually employed to measure the concentration of proteins in biological fluids because, based on the few available information regarding AH proteins, low protein concentrations are expected (range 22 to 75 mg/dL from [17]) and the lower limit of quantification of the biuret method would not be sufficient.

### 2.5. RT-nPCR for FCoV

From frozen-thawed AH samples, RNA was obtained using a NucleoSpin RNA kit (Macherey-Nagel, Bethlehem, PA, USA) according to the manufacturer’s instructions. RNA samples were then frozen at −80 °C or immediately used for PCR. A reverse transcription nested PCR (RT-nPCR) targeting a 177 bp product of the highly conserved 3′ untranslated region (3′ UTR) of the genome of both type I and type II FCoV was used [18]. FCoV RT-nPCR positive RNA was used as the positive control and RNase-free water as the negative control. PCR products were visualized under a UV transilluminator on a 1.5% agarose gel stained with ethidium bromide. The PCR was considered positive when showing a 177 bp band in the second round RT-PCR on agarose gel electrophoresis. A subset of positive RT-PCR samples of cats with FIP was analyzed for the presence of a positive 233 bp band in the first round RT-PCR on agarose gel electrophoresis [18].

### 2.6. Post Mortem Examination and Group Formation

Necropsies were performed within 12 h of death, on remains kept either refrigerated or at room temperature. The specimens from the organs either affected by gross lesions or possibly affected based on clinical history (e.g., liver, kidney, lymph nodes) were collected, fixed in 10% buffered formalin and paraffin embedded. Histology was performed on 5-μm sections stained with hematoxylin and eosin. IHC for FCoV was performed using a mouse monoclonal antibody anti-FCoV (FIPV3-70 clone, Serotec, Bio-Rad, Segrate, Italy) using protocols already described [9,19].

Cats were assigned to the FIP group if histopathologic findings revealed typical lesions along with positive immunohistochemistry (IHC) in one or more of the examined tissues (an example of positive immunohistochemistry is provided in Figure S1). For statistical purposes, this group was further divided into effusive FIP (E-FIP) and non-effusive FIP (NE-FIP), based on the presence or absence of cavitary effusions, respectively. Cats were assigned to the non-FIP group based on history, laboratory and/or diagnostic findings and histology revealing diseases other than FIP.

### 2.7. Statistical Analyses

Statistical analysis was run with the Analyse-it software v. 6.15.4 (Analyse-it Ltd., Leeds, UK). A non parametric *t*-test for independent data (Wilcoxon–Mann–Whitney test) was used to compare the age range of FIP vs. non-FIP cats and a non parametric ANOVA test for independent data (Kruskall–Wallis test) was used to compare the age range of E-FIP, NE-FIP and non-FIP cats. The differences in the AH total protein concentrations in FIP vs. non-FIP cats, in cats with positive or negative PCR results, and in cats with cytology consistent or not with FIP were evaluated using a Wilcoxon–Mann–Whitney test. The difference in AH total protein concentration among non-FIP cats, cats with effusive FIP and cats with non-effusive FIP was assessed using the Kruskall–Wallis test, followed by the Wilcoxon–Mann–Whitney test for the comparison of pairs of groups.

A Pearson chi-squared test was used to evaluate the differences in the proportion of positive cytology and PCR results in FIP or non-FIP cats.

In order to assess the diagnostic performance of results reported as positive or negative (RT-PCR, cytological patter, presence of proteinaceous background), the number of true positive (TP = cats with FIP and with positive), false positive (FP = cats without FIP with positive results), true negative (TN = cats without FIP with negative results), and false negative (FN = cats with FIP with negative results) was calculated. Based on the number of TP, FP, TN, and FN, sensitivity (Sens) and specificity (Spec) were calculated using standard formulae [20]. Using Sens and Spec, the positive likelihood ratio (LR+) was calculated using the formula LR+ = Sens/(1-Spec), and the negative likelihood ratio (LR−) calculated using the formula LR− = (1-Sens)/Spec.

## 3. Results

### 3.1. Caseload and Samplings

Overall, 44 cats were included in the study. Data regarding the signalment of each cat and group assignment are reported in Table 1, along with the results recorded in the different tests.

A total of 28 cats were affected by FIP as confirmed by post-mortem histopathology and IHC gold standard diagnosis (13 were allocated in the E-FIP group, 15 in the NE-FIP group), while 16 cats were assigned to the NON-FIP group. The most common breed was the domestic shorthair (DSH, 33 cats), followed by the Maine Coon and the Abyssinian (3 cats each) and by Bengala, Norwegian Forest, Russian Blue, Siberian and Sphynx, each one represented by 1 subject. The ages ranged from 3 months to 10 years in FIP cats (median 9 months), with slightly older cats in the NE-FIP group (median 12 months), compared with E-FIP cats (median 6 months). Non-FIP cats’ age ranged between 4 months and 14 years (median 2 years). No significant differences were found regarding age, or between FIP and non-FIP cats nor among the subgroups. Based on the history and histopathological results, cats from the non-FIP group were affected by infectious diseases (feline panleukopenia, salmonellosis and bacterial pneumonia with septicemia in cats n° 29, 35 and 41, respectively); neoplastic diseases (meningioma, malignant histiocytosis and sarcoma in cats n° 31, 32, and 38, respectively); severe hepatic lipidosis (cats n° 36 and 40); multiple traumatic injuries (cats n° 42 and 43); chronic kidney disease (cats n° 37 and 39) and miscellaneous diseases (hereditary amyloidosis, necrotizing encephalitis, severe malnutrition and massive helminths infestation in cats n° 30, 33, 34 and 44, respectively).

Along with the symptoms usually described in FIP, such as fever, anorexia, lethargy and jaundice, some of the FIP affected cats also presented neurological and/or ocular signs in vivo. In particular, cat n° 11 of the E-FIP group had macroscopic signs of uveitis in the left eye. In the NE-FIP group, cats n° 14, 15, 17, 20, 22, and 23 had neurological signs (i.e., seizures, ataxia, paralysis), while cat n° 19 had neurological signs and gross lesions consistent with uveitis and cats n° 18 and 24 had signs of uveitis alone. In the non-FIP group, cats n° 31 and 33 were described as having neurological signs (ataxia and seizures, respectively).

When possible, aqueous humor was collected from both eyes. Overall, cytology was performed on 83 samples, protein content measurement on 80 samples and RT-PCR for FCoV on 66 samples (see Table 1). The subset of RT-PCR positive samples of cats with FIP showed negative results in the first round of the RT-PCR.

### 3.2. Frequency of Positive Results and Comparison of Protein Content

Results regarding the data recorded in each group or subgroup are summarized in Table 2.

No significant differences were found in the proportion of RT-PCR positive samples between the whole FIP group and the non-FIP group (*p* = 0.344) or between the E-FIP group and the NE-FIP group (*p* = 0.526). In 30 cats (20 with FIP and 10 without FIP), RT-PCR was performed in both eyes and in 7 out of these 30 cases (23.3%) the results were discordant between the two eyes, having positive results in only one of the two AH samples analyzed.

**Figure 1:**
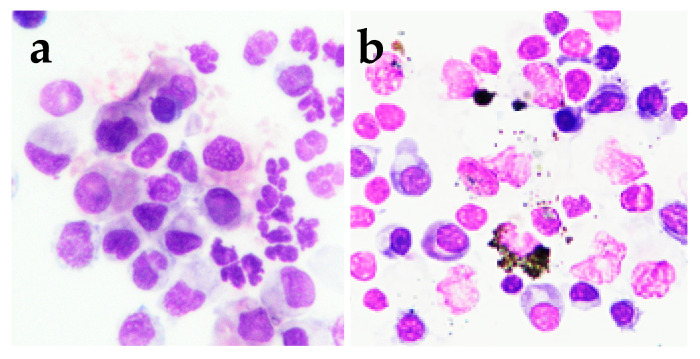
Examples of positive cytological results detectable on May–Grunwald–Giemsa stained cytocentrifuged cytological specimens of cats with FIP (1000× magnification). (**a**) Cluster of cells composed by non degenerated neutrophils, small lymphocytes and macrophages; (**b**) cytological specimen characterized mostly by small lymphocytes and plasma cells, and by occasional melanin-containing cells.

Regarding cytology, all the samples were of good quality (few to none broken cells, debris or other artifacts that may occur in samples collected post-mortem were found) and the majority of samples were either acellular or with very rare corneal, mononuclear and/or red blood cells. Positive samples were more frequent in the FIP group than in the non-FIP group (*p* = 0.042), but no significant differences were found between the proportion of positive samples in the FIP subgroups (*p* = 0.126). In most of the positive cases the cellular population was mixed, with neutrophils (mostly non degenerated), lymphocytes and monocytoid cells (Figure 1), although a pure population of neutrophils (seven samples) or, more rarely, of lymphocytic and monocytoid cells (two cases) were sometimes found.

**Figure 2:**
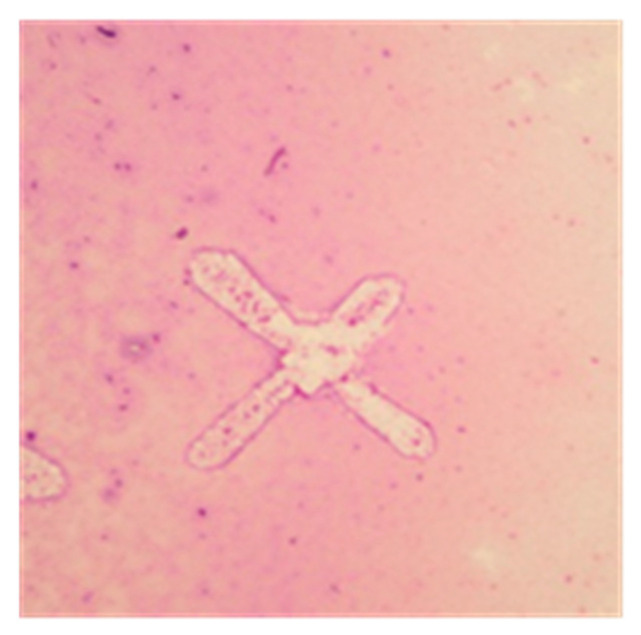
Example of proteinaceous background detectable on cytocentrifuged cytological specimens of cats with FIP (May–Grunwald–Giemsa stain, 100× magnification): crystal-like structures detectable in samples characterized by abundant granular and diffuse eosinophilic proteinaceous background.

When present, the proteinaceous, eosinophilic background, was characterized by scattered granular material, occasionally abundant and thick, embedding the cells, if present, or including regularly shaped structures likely due to the crystallization of salts present on the fluid (Figure 2). This proteinaceous background was observed, with similar proportions, in samples from cats with and without FIP (*p* = 0.316), and in samples from the two subgroups of cats with FIP (*p* = 0.483).

In 40 cats (27 with FIP and 13 without FIP), cytology was performed in both eyes, and discordant results between the two eyes with positive results in only one of the two AH samples analyzed were found in six cases (15.0%) for the cytological pattern, and in eight cases (20.0%) for the proteinaceous background.

Cytology was more frequently (*p* = 0.007) positive in samples with positive RT-PCR (11/22, 50.0%) than in samples that were RT-PCR negative (8/44, 18.2%), likely due to the samples from FIP cats, on which the proportion of positive cytological samples was higher (*p* = 0.009) in samples with positive RT-PCR (10/17, 58.8%) than in those with negative RT-PCR (6/29, 20.7%). Conversely, no significant differences (*p* = 0.718) were found among samples from non-FIP cats on which 1/5 (20.0%) of the RT-PCR positive samples had positive cytology, while 2/15 (13.3%) of the RT-PCR negative samples had positive cytology (71.8%). The presence of the proteinaceous background was recorded in the same proportion in samples with a positive cytology (10/20, 50.0%) and in samples with a negative cytology (26/63, 46.03%, *p* = 0.757) or in samples with a positive RT-PCR (8/22, 36.4%) and with a negative RT-PCR (20/44, 45.5%, *p* = 0.481).

**Figure 3:**
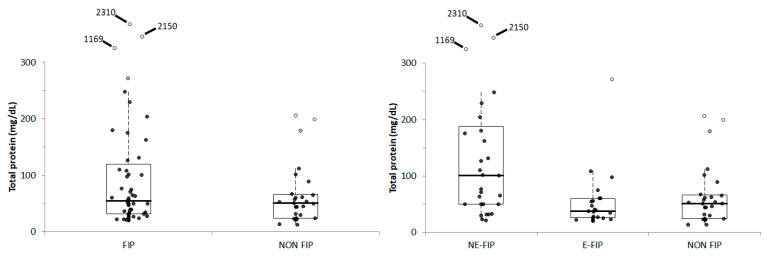
Protein concentration recorded in cats with or without FIP (FIP and non-FIP, respectively) or in cats with non-effusive FIP (NE-FIP) and effusive FIP (E-FIP) compared with cats without FIP (NON-FIP). The boxes indicate the I-III interquartile range (IQR); horizontal lines indicate the median value. Vertical lines extend until the last value not classifiable as outlier. Black circles indicate the results not classifiable as outliers; gray circles and open circles indicate, respectively, the near outliers (values higher than the III quartile + 1.5 × IQR) and the far outliers (values higher than the III quartile + 3.0 × IQR). Far outliers exceeding the scalebar are reported on the top of each graph.

The protein concentration (Figure 3) ranged from 20.0 to 272.0 mg/dL (mean ± S.D. 53.6 ± 53.3 mg/dL; median 36.9 mg/dL) in E-FIP samples, from 20.9 to 2310.0 mg/dL (mean ± S.D. 300.6 ± 582.6 mg/dL; median 101.0 mg/dL) in NE-FIP samples and from 12.9 to 205.9 mg/dL (mean ± S.D. 63.1 ± 52.8 mg/dL, median 50.7 mg/dL) in non-FIP samples. Overall, no significant differences (*p* = 0.207) were found between non-FIP samples and the whole group of FIP samples (range: 20.0–2310.0 mg/dL; mean ± S.D. 191.3 ± 450.4 mg/dL; median 55.2 mg/dL), but when the two FIP subgroups were considered separately a significant difference among groups was found (*p* = 0.017), with higher values in NE-FIP samples compared with both E-FIP samples (*p* = 0.001) and non-FIP samples (*p* = 0.008).

In two NE-FIP cats, for a total of three samples, very high protein concentrations were recorded. These samples were the AH from the right eye of cat n° 17 (1169.5 mg/dL), and both the AH of cat n° 18 (2310 and 2150 mg/dL).

On the whole caseload, the protein concentration was significantly higher in PCR-positive samples (20.9–2310.0 mg/dL; mean ± S.D. 302.5 ± 644.1 mg/dL; median 97.5 mg/dL) than in PCR-negative samples (20.0–672 mg/dL; mean ± S.D. 84.1 ± 111.0 mg/dL; median 49.5 mg/dL) (*p* = 0.005) and in samples with positive cytology (24.5–2310.0 mg/dL; mean ± S.D. 353.0 ± 687.2 mg/dL; median 106.3 mg/dL) than in samples with negative cytology (12.9–1169.5 mg/dL; mean ± S.D. 87.8 ± 169.8 mg/dL; median 46.2 mg/dL) (*p* < 0.001). Conversely, no significant differences were found between samples with a proteinaceous background (12.9–2310.0 mg/dL; mean ± S.D. 235.0 ± 521.1 mg/dL; median 52.9 mg/dL) and samples without a proteinaceous background (20.0–272.0 mg/dL; mean ± S.D. 67.2 ± 52.3 mg/dL; median 53.3 mg/dL) (*p* = 0.603).

The proportions of positive cats obtained using this approach in groups and subgroups of cats are also reported in Table 2. As stated above, the diagnostic potential of RT-PCR and of both the cytologic findings (inflammatory pattern and proteinaceous background) was assessed only on cats on which each test was performed in both eyes, and for each test cats that had at least one positive result were considered as “positive”, while cats with negative results in both eyes were considered as “negative”. Significant differences in terms of the proportion of cats positive in at least one eye were never detected between FIP and non-FIP cats (positive PCR: *p* = 0.196; positive cytology: *p* = 0.109; presence of proteinaceous background: *p* = 0.145) or between subgroups of FIP cats (positive PCR: *p* = 0.582; positive cytology: *p* = 0.310; presence of proteinaceous background: *p* = 0. 883). In cats with FIP, according to available information on the presence of clinical signs other than effusion, RT-PCR was positive in all four cats with FIP showing uveitis whereas RT-PCR positive results were obtained in the minority (16.7%) of cats with neurological signs only.

### 3.3. Diagnostic Performances

The sensitivity, specificity and likelihood ratios of each test are reported in Table 3.

All the tests have moderate to high specificity, with cytology performing better than the PCR and proteinaceous backgrounds, especially in terms of a positive likelihood ratio. The specificity was even higher when PCR and cytology were considered together. However, the sensitivity was low for all the tests, with the negative likelihood ratio largely being far from the optimal value of 0.00.

## 4. Discussion

The aim of this study was to evaluate the utility of clinicopathological and molecular findings on the aqueous humor (AH) for the diagnosis of FIP. The diagnosis of FIP is still challenging, especially in the non-effusive form, which often manifests itself with ocular signs such as uveitis, and which lacks a very useful diagnostic matrix as the effusion [1,13]. In the current study, cats with FIP were grouped according to the conventional classification based on the presence of the effusions, although the current guidelines do not emphasize classifying E-FIP and NE-FIP and classification based on clinical signs (pleural effusion, ascites, uveitis, and neurological sign) may be proposed [21]. However, in each single cat with FIP, lesions (and associated signs) may be present in more than one organ and therefore a complete classification based on clinical signs was not performed in this study because it would have led to several groups composed of very low number of animals.

In particular, the study was focused on RT-PCR for the FCoV and, as regards clinico-pathological testing, on the measurement of protein content and on the cytological appearance of cytocentrifuged smears, with special emphasis on two main aspects that can be frequently found in ocular fluids, namely the presence of an inflammatory cell population (neutrophils and/or lymphocytes and/or macrophages) and the presence of precipitated proteins on the background [10,11]. To assess the diagnostic performances of all these tests, we first evaluated the frequency of positive results (i.e., positive RT-PCR or samples with cytology consistent with inflammation or characterized by the proteinaceous background) in AH samples collected from cats with or without FIP, as well as the possible difference in the concentration of total proteins between these groups. Then, based on the results from cats that were sampled in both eyes, the diagnostic performance of RT-PCR and of cytological patterns in detecting cats with FIP with at least a positive result in one of the eyes was evaluated in terms of sensitivity, specificity and likelihood ratios. All the tests above were performed irrespective of the clinical presentation (i.e., presence or absence of ocular signs) or of the detection of macroscopical or microscopical lesions, assuming that AH may contain the virus and possible associated clinico-pathological changes even in the absence of these lesions, due to the vasculitis that may characterize FIP.

However, this approach demonstrated that all the changes above are not constantly present in samples from cats with FIP and, conversely, may be detected also in cats with diseases other than FIP and, consequently, the diagnostic parameters of all the tests to support a clinical diagnosis of FIP are not as high as other tests commonly recommended in the diagnostic approach to FIP [1,3,9]. On the other hand, the RT-PCR positive results in cats with FIP showing uveitis may suggest AH analysis as an effective diagnostic method for cats with signs of uveitis, but the results need to be confirmed by further investigation based on larger caseloads.

The negative RT-PCR results in the AH of cats with FIP is not surprising, since it is known that this test is not particularly sensitive even in the presence of ocular lesions, on which the reported sensitivity for RT-PCR is 50% [14]; the PCR targeting all FCoVs and the mutated S gene, which were not tested in the current study, were positive in 25% and 10% of the FIP cats, respectively [15]. Therefore, it could be expected that sensitivity equals, or is even lower, in cats sampled, irrespective of the presence of ocular signs. Conversely, the detection of samples positive to RT-PCR in the non-FIP group is quite surprising since it indicates that FCoVs may be present in the AH even in non-FIP affected cats. Previous studies have shown that FCoV has been detected in organs of both FIP and non-FIP cats [13]. It can be presumed that cats with positive RT-PCR in the AH had circulating FCoVs and that the blood–ocular barrier was somewhat damaged or permeabilized. Unfortunately, information on molecular positivity in blood of cats sampled in this study was not always available. However, the diseases of the three cats from which the five RT-PCR positive samples were collected (two chronic kidney diseases, one polytrauma) are not known to increase the permeability of the ocular barrier and therefore the reason of such a high rate of positive samples compared with previous studies remains to be elucidated. Independent of the mechanism responsible for this positivity, the detection of positive cats in the non-FIP group reduces the specificity of the test, that in previous studies, based on samples from cats with or without ocular lesions, has been reported to be as high as 100% [16].

The detection of an inflammatory pattern in cytological specimens in our caseload was much more specific than RT-PCR, although less sensitive. The few reports on cytological techniques on ocular fluid do not provide exhaustive information on the diagnostic performances of routine cytology [10,12], or indicate that cytology may be not specific enough to diagnose FIP [22]. Conversely, immunocytochemistry on AH specimens has been shown to support a clinical diagnosis of FIP [12,16]. Based on the cited reports, however, the specificity of immunohistochemistry (around 80%), was even lower than that recorded in the current study for cytology alone, but the sensitivity was higher (more than 60%). Also, in the case of cytology, positive results, i.e., the detection of inflammatory patterns, were found also in samples from non-FIP cats, likely depending on the type of disease with positive cytological results in cats from the non-FIP group. However, the positive association between inflammatory patterns in cytological specimens and RT-PCR suggests that the presence of the virus may play a role in inducing an inflammatory profile in the AH, as demonstrated also in histological studies in which both the virus and the inflammatory cells have been immunohistochemically characterized [23]. In support of this hypothesis, the diagnostic performance further increases when cytology and PCR are considered together. Ultimately, based on the positive likelihood ratio, the probability that a cat with positive cytology or positive cytology and PCR in at least one eye is affected by FIP is, respectively, 2.65 times and 3.48 times higher than the probability that this cat has another disease. However, based on the low sensitivity and the high negative likelihood ratio, it cannot be excluded that a cat with negative cytology and/or PCR in both eyes is affected by FIP.

The cytological examination of ocular fluids also revealed the presence of a proteinaceous background, which was also mentioned in another study [12] but not characterized in terms of frequency in different pathologic conditions, including FIP. The current study, however, demonstrated that this background can be found almost in equal proportions, in samples from cats with and without FIP and therefore it appears to be poorly sensitive and specific and ultimately not associated with the presence of cytological patterns consistent with inflammation nor with positive RT-PCR results. Conversely, samples with this proteinaceous background had a higher concentration of protein in AH compared with samples that did not have an evident proteinaceous background, suggesting that this cytological finding is a consequence of the higher protein content, independent of the cause of the increased concentration of protein.

The measurement of total protein in the AH seems to be a promising tool for supporting a diagnosis of FIP, since the protein concentrations were significantly higher in samples from cats with FIP than in samples from cats with non-FIP, and in particular in samples from cats with NE-FIP than in samples from cats with E-FIP. Moreover, the higher protein concentration in samples with positive RT-PCR or with cytological patterns consistent with inflammation suggest an association among these findings that deserves to be investigated further through future studies. Unfortunately, reports about the normal concentration of protein in the ocular fluids of cats is lacking and, on the one hand, it was difficult to establish whether the magnitude of the concentrations recorded in this study is sufficiently high compared with normal values to be “per se” diagnostic; on the other hand, it was not possible to dichotomize the results in positive or negative and it was therefore not possible to classify cats as positive or negative based on a positive protein result in at least one eye. Hence, it was not possible to calculate sensitivity, specificity and likelihood ratios for the concentration of proteins.

Our results, however, suggest that the protein content in the ocular fluids of sick cats may be very high, as recorded in some cats, on which the protein content was higher than 1000 and sometimes 2000 mg/dL. On the one hand, this may depend on the progressive accumulation of proteins in the anterior chamber, but on the other hand, the possible presence of preanalytical artifacts due to a sort of matrix effect of the ocular fluid or of other interferents that may accumulate in the eye (e.g., bilirubin in the sample with the highest protein concentration that was collected from an icteric cat and was macroscopically yellowish) cannot be excluded. Additionally, in the current study, pyrogallol red has been used to measure the concentration of proteins assuming that the protein content of the fluid was low, and it may be possible that very high values are inaccurate since this reagent lacks linearity at high protein values [24] However, despite previous reports where the measurement of intraocular proteins was considered not specific enough [22], our results suggest that this test, contrarily to the other included in the current study, may have some utility in the diagnostic approach to FIP cases.

This study has some limitations. Firstly, information on the actual presence of FIP lesions or of inflammation in the eyes of sampled cats was not available since the cats were not visited by an ophthalmologist and histopathology was not performed in all cases, due to the impossibility to perform post-mortem analyses in a timely manner and while avoiding storage artifacts. However, the analysis of samples collected, irrespective of the presence of lesions, better simulates what may happen in routine practice “in vivo”, where the sampling of ocular fluid is usually recommended by ophthalmologists based on clinical findings and not on the actual detection of ocular histological lesions. Secondly, no information was available on the possible presence of viral antigen in the blood, and therefore it is not possible to conclude whether the viral genome detected in the AH originated from blood after the disruption of the hemato–ocular barrier or was released locally from inflammatory sites. Thirdly, the number of NE-FIP cases was too low to assess whether the tests included in this study may be useful specifically in the diagnosis of dry FIP. Lastly, real-time reverse-transcriptase-PCR (RT-qPCR), that is known to be more sensitive than conventional RT-nPCR, was not performed and therefore false negative results may not be ruled out in this study [25]. RT-qPCR should be used in the diagnosis of FIP that relies on the detection of high FCoV RNA load, rather than proving the presence of FCoV. Moreover, the virus in the RT-PCR positive samples was not genotyped and information on the presence of the FCoV type I and FCoV type II was not available from this study.

## 5. Conclusions

In conclusion, the current study provided useful information on the use of AH as a surrogate matrix to be included in the diagnostic approach to FIP cases. In particular, compared with the few studies so far published on ocular fluids of cats with FIP, this is the first report that combines molecular and clinico-pathological findings to provide a comprehensive overview on the diagnostic potential of this specimen. Ultimately, none of the tests that can dichotomize the results as “positive” or “negative” (RT-PCR, cytology consistent with inflammation or evidencing a proteinaceous background) seem to be sensitive or specific enough to be recommended as an ancillary test to support a diagnosis of FIP. Among these tests, however, routine cytology seems to be the most specific, and based on its moderately high LR+, this may further increase if associated with the results of RT-PCR, potentially working as a supportive test when the pre-test probability of FIP is high according to a Bayesian approach [26]. Conversely, the concentration of intraocular protein seems to be a promising marker in differentiating cats with FIP, especially in the non-effusive form. This latter aspect merits further exploration through future studies, aimed first to define the normal protein content of ocular fluid and some analytical aspects such as those associated with the use of pyrogallol compared to other reagents, and then to determine the diagnostic potential of protein measurement on a larger caseload with more strict inclusion criteria (also based on the histological or immunohistochemical characterization of ocular tissues), using a higher proportion of non-FIP cats with clinical presentations potentially confounding with FIP, especially in its dry form.

## Figures and Tables

**Table 1:** Signalment of the cats enrolled in the study and results recorded in each specimen.

	Group	Signalment	Left Eye				Right Eye			
			Cytology		Protein (mg/dL)	RT-PCR	Cytology		Protein (mg/dL)	RT-PCR
			Cells	Background			Cells	Background		
1	E-FIP	MC, M, 5 m	Neg	Neg	39.8	ND	Neg	Pos	36.8	ND
2	E-FIP	MC, M, 3 m	Neg	Pos	26.8	ND	Neg	Neg	25.9	Neg
3	E-FIP	DSH, Unk, 7 m	Neg	Pos	39.4	ND	Neg	Pos	22.5	ND
4	E-FIP	DSH, Unk, 4 m	Neg	Pos	20.9	Neg	Neg	Pos	22.0	ND
5	E-FIP	Aby, Unk, Unk	Neg	Neg	36.9	Neg	Neg	Neg	59.9	Neg
6	E-FIP	DSH, M, 3 m	Neg	Pos	55.4	Pos	Neg	Neg	97.5	Pos
7	E-FIP	DSH, M, 1 y	Pos	Pos	24.5	Pos	Pos	Pos	28.1	Neg
8	E-FIP	DSH, M, 6 m	Neg	Pos	25.3	Neg	Neg	Pos	ND	Neg
9	E-FIP	Ben, M, 6 m	Neg	Neg	34.6	Neg	Neg	Neg	47.0	Neg
10	E-FIP	Sph, MN, 10 y	Neg	Neg	108.2	Pos	Pos	Pos	74.6	Neg
11	E-FIP	MC, FN, 18 m	Pos	Neg	ND	Pos	Pos	Neg	ND	ND
12	E-FIP	DSH, FN, 5 m	Pos	Neg	55.0	Pos	Pos	Neg	60.0	Pos
13	E-FIP	DSH, F, 9 m	Neg	Neg	20.0	Neg	Pos	Neg	272.0	Pos
14	NE-FIP	DSH, Unk, Unk	Neg	Pos	49.7	ND	Neg	Neg	49.6	Neg
15	NE-FIP	DSH, M, 6 m	Neg	Pos	32.0	Neg	Neg	Pos	31.5	Neg
16	NE-FIP	DSH, M, 1 y	Neg	Pos	162.3	Neg	ND	ND	ND	ND
17	NE-FIP	DSH, MN, 3 y	Neg	Pos	672.0	Neg	Neg	Pos	1169.5	ND
18	NE-FIP	NF, M, 14 m	Pos	Pos	2310	Pos	Pos	Pos	2150	Pos
19	NE-FIP	Aby, M, 7 m	Pos	Neg	49.5	Neg	Pos	Neg	110.6	Pos
20	NE-FIP	DSH, M, Unk	Neg	Pos	32.4	Neg	Neg	Pos	20.9	Pos
21	NE-FIP	DSH, M, 1 y	Neg	Pos	126.3	Pos	Pos	Pos	131.0	Pos
22	NE-FIP	DSH, M, 1 y	Neg	Neg	ND	Neg	Neg	Neg	65.5	Neg
23	NE-FIP	DSH, F, 10 m	Neg	Neg	63.1	Neg	Neg	Neg	175.3	Neg
24	NE-FIP	DSH, FN, 1 y	Neg	Neg	203.9	Pos	Neg	Pos	101.7	Neg
25	NE-FIP	DSH, F, 5 y	Pos	Pos	180.4	Pos	Neg	Neg	76.6	Pos
26	NE-FIP	DSH, FN, 3 y	Pos	Pos	229.5	Neg	Neg	Pos	248.2	Neg
27	NE-FIP	DSH, M, 5 m	Neg	Neg	30.0	Neg	Neg	Neg	23.0	Neg
28	NE-FIP	DSH, F, 6 m	Pos	Neg	101.0	Neg	Pos	Neg	71.0	Neg
29	NON-FIP	DSH, F, Unk	ND	ND	ND	ND	Neg	Pos	31.9	ND
30	NON-FIP	Aby, F, 4 y	ND	ND	ND	ND	Neg	Pos	50.4	ND
31	NON-FIP	DSH, FN, 9 y	Neg	Pos	12.9	ND	Neg	Pos	13.2	ND
32	NON-FIP	DSH, FN, Unk	Neg	Pos	179.0	Neg	Neg	Neg	45.5	Neg
33	NON-FIP	DSH, FN, 1 y	Neg	Neg	112.2	ND	Neg	Neg	23.5	ND
34	NON-FIP	Sib, F, 6 m	Neg	Neg	21.7	Neg	Neg	Neg	22.3	Neg
35	NON-FIP	RB, M, 4 m	Neg	Pos	66.9	Neg	Neg	Pos	57.4	Neg
36	NON-FIP	DSH, Unk, Unk	Neg	Pos	60.9	Neg	Neg	Pos	30.1	Neg
37	NON-FIP	DSH, MN, 11 y	Neg	Neg	53.7	Neg	Neg	Neg	61.8	Pos
38	NON-FIP	DSH, Unk, Unk	Pos	Pos	205.9	Neg	Pos	Pos	199.2	Neg
39	NON-FIP	DSH, MN, 14 y	Neg	Neg	65.0	Pos	Neg	Neg	53.0	Pos
40	NON-FIP	DSH, F, 3 y	ND	ND	51.0	ND	ND	ND	44.0	ND
41	NON-FIP	DSH, F, Unk	Neg	Neg	23.0	Neg	Neg	Neg	44.0	Neg
42	NON-FIP	DSH, F, Unk	Pos	Neg	102.0	Pos	Neg	Neg	89.0	Pos
43	NON-FIP	DSH, M, 1 y	Neg	Neg	ND	ND	Neg	Neg	ND	ND
44	NON-FIP	DSH, F, 6 m	Neg	Neg	24.0	Neg	Neg	Neg	23.0	Neg

E-FIP: effusive FIP; NE-FIP: non-effusive FIP; MC: Maine Coon; DSH: domestic shorthair; Aby: Abyssinian; Ben: Bengala; Sph: Sphynx; NF: Norwegian Forest; Sib: Siberian; RB: Russian Blue; M: Male; F: Female; MN: neutered male; FN: neutered female; Unk: unknown; y: years; m: month; Pos: positive; Neg: negative; ND: not done.

**Table 2:** Frequency of positive results recorded for each test on the total number of samples or on the total number of cats. The percentage of positive samples or cats is also reported in brackets.

		Groups		Subgroups	
		FIP	Non-FIP	E-FIP	NE-FIP
RT-PCR	Positive samples	17/46 (37.0%)	5/20 (25%)	8/19 (42.1%)	9/27 (33.3%)
	Positive cats	11/20 (55.0%)	3/10 (30.0%)	5/8 (62.5%)	6/12 (50.0%)
Cytology	Positive samples	17/55 (30.8%)	3/28 (10.7%) *	8/26 (30.8%)	9/29 (31.0%)
	Positive cats	11/27 (40.7%)	2/13 (15.4%)	4/13 (30.8%)	7/14 (50.0%)
Proteinaceous background	Positive samples	28/55 (50.9%).	11/28 (38.3%)	12/26 (46.2%)	16/29 (55.2%)
	Positive cats	17/27 (63.0%)	5/13 (38.5%)	8/13 (61.5%)	9/14 (64.3%)

* *p* < 0.05 compared with FIP cats.

**Table 3:** Diagnostic performances of the different test to support a clinical diagnosis of FIP.

	Sensitivity	Specificity	LR+	LR−
RT-PCR	55.0%	70.0%	1.83	0.64
Cytology	40.7%	84.6%	2.65	0.70
Proteinaceous background	63.0%	61.5%	1.64	0.60
RT-PCR and Cytology	34.8%	90.0%	3.48	0.72

LR+ = positive likelihood ratio; LR− = negative likelihood ratio.

## Data Availability

All the data generated within this study are reported in Table 1.

## Supplementary Materials

The following supporting information can be downloaded at: https://www.mdpi.com/article/10.3390/vetsci11050207/s1, Figure S1: Exemplificative images of positive results in FIP cats.
