# Supplementary material for: Clinicopathological and Molecular Analysis of Aqueous Humor for the Diagnosis of Feline Infectious Peritonitis
Source: Vet Sci. 2024 May 9;11(5):207. doi: 10.3390/vetsci11050207 (PMC11125769; doi:10.3390/vetsci11050207)

Supplementary figure

Exemplificative images of positive results in FIP cats. A) multifocal pyogranulomatous lesions in the omentum (Hematoxylin and eosin stain, 40X magnification); B) scattered FCoV-positive cells within the pyogranulomatous lesions (anti-FCoV immunohistochemistry, Immunoperoxidase staining). Histological and immunohistochemical details are reported at higher magnification (100X) n the inserts at the left bottom corner of each image.

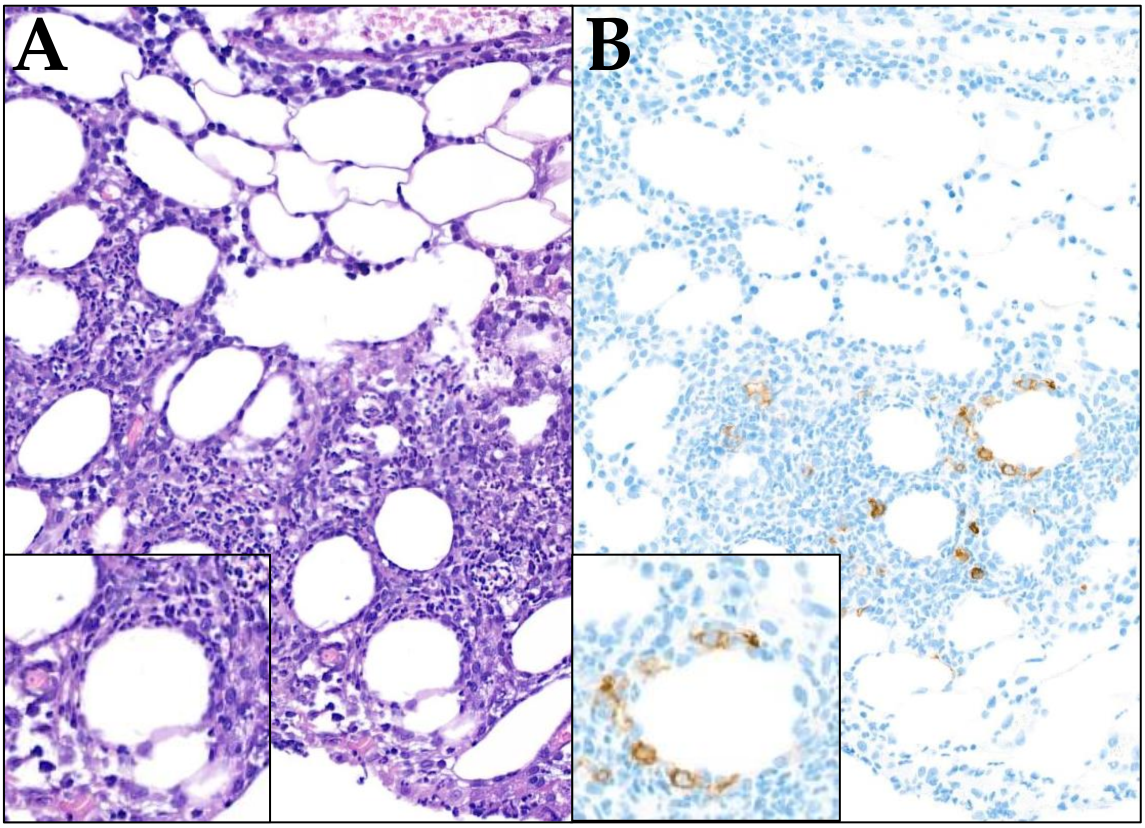

Supplement: Supplementary file 1 [file vetsci-11-00207-s001.zip › vetsci-2937438-supplementary.pdf]
